# The Coronary Angiography-Derived Index of Microcirculatory Resistance Predicts Left Ventricular Performance Recovery in Patients with ST-Segment Elevation Myocardial Infarction

**DOI:** 10.1155/2022/9794919

**Published:** 2022-07-14

**Authors:** Chang Hou, Meng Guo, Yuliang Ma, Qi Li, Chuanfen Liu, Mingyu Lu, Hong Zhao, Jian Liu

**Affiliations:** ^1^Department of Cardiology, Peking University People's Hospital, Beijing, China; ^2^Beijing Key Laboratory of Early Prediction and Intervention of Acute Myocardial Infarction, Peking University People's Hospital, Beijing, China

## Abstract

**Objectives:**

The present study is designed to investigate the impact of coronary angiography-derived index of microcirculatory resistance (caIMR) on left ventricular performance recovery.

**Background:**

IMR has been established as a gold standard for coronary microvascular assessment and a predictor of left ventricular recovery after ST-segment elevation myocardial infarction (STEMI). CaIMR is a novel and accurate alternative of IMR.

**Methods:**

The present study retrospectively included 80 patients with STEMI who underwent primary percutaneous coronary intervention (PCI). We offline performed the post-PCI caIMR analysis of the culprit vessel. Echocardiography was performed within the first 24 hours and at 3 months after the index procedure. Left ventricular recovery was defined as the change in left ventricular ejection fraction (LVEF) more than zero.

**Results:**

The mean age of the patients was 58.0 years with 80.0% male. The average post-PCI caIMR was 43.2. Overall left ventricular recovery was seen in 41 patients. Post-PCI caIMR (OR: 0.948, 95% CI: 0.916–0.981, *p* = 0.002), left anterior descending as the culprit vessel (OR: 3.605, 95% CI: 1.23–10.567, *p* = 0.019), and male (OR: 0.254, 95% CI: 0.066–0.979, *p* = 0.047) were independent predictors of left ventricular recovery at 3 months follow-up. A predictive model was established with the best cutoff value for the prediction of left ventricular recovery 2.33 (sensitivity 0.610, specificity 0.897, and area under the curve 0.765). In patients with a predictive model score less than 2.33, the LVEF increased significantly at 3 months.

**Conclusions:**

The post-PCI caIMR can accurately predict left ventricular functional recovery at 3 months follow-up in patients with STEMI treated by primary PCI, supporting its use in clinical practice.

## 1. Introduction

Although primary percutaneous coronary intervention (PCI) can restore the blood flow of the epicardial coronary artery, coronary microvascular dysfunction (CMD) still exists in patients with ST-segment elevation myocardial infarction (STEMI) [1]. The post-PCI hyperemic index of microcirculatory resistance (IMR) obtained by pressure wire is a useful tool for the assessment of CMD and can effectively predict left ventricular recovery at 3 months post-STEMI [2–4]. However, the clinical adoption of IMR remains limited mainly due to additional cost and procedural complexity.

Coronary angiography-derived IMR (caIMR) is an emerging computed index to evaluate coronary microcirculation without physiology wire and adenosine [5], which shows accurate diagnostic performance for CMD and great long-term prognostic value in previous studies [6–15]. However, the impact of post-PCI caIMR on left ventricular performance recovery at 3 months in patients with STEMI remains unknown. The aim of the present study is to examine whether caIMR can predict left ventricular recovery at 3 months after the index procedure.

## 2. Materials and Methods

### 2.1. Study Population

The present study consecutively enrolled 134 patients with STEMI who underwent primary PCI at Peking University People's Hospital (Beijing, China) between July 2016 and December 2021. STEMI was defined using the fourth universal definition of myocardial infarction [16]. The exclusion criteria were as follows: merely coronary angiography without stent implantation, coronary artery bypass graft rather than PCI, lack of echocardiography within the first 24 hours and after 3 months, and poor angiographic image quality precluding the contour detection and caIMR calculation.

### 2.2. Study Design

This was a retrospective study to evaluate the predictive ability of post-PCI caIMR on left ventricular performance recovery at 3 months in patients with STEMI. We searched and reviewed medical records, coronary angiography, and echocardiography images of the eligible patients. The patient's demographic information, cardiovascular risk factors, hemodynamic parameters, laboratory examinations, discharge medications, lesion, and procedural characteristics were recorded.

#### 2.2.1. PCI Procedure and Coronary Microcirculation Assessment

Coronary angiography and stent implantation were performed according to the standard protocol. An image acquisition speed of 30 frames per second was used. The thrombolysis in myocardial infarction (TIMI) flow grade was assessed in all patients. Corrected TIMI frame count (cTFC) was calculated as previously described [17]. The caIMR analysis of infarction related artery (IRA) after PCI was achieved offline by using commercialized software (FlashAngio, Rainmed Ltd., Suzhou, China) as described in literature [8]. In brief, a three-dimensional reconstruction was first conducted for the interrogated vessel; then coronary angiography-derived fractional flow reserve (caFFR) was estimated by computational pressure-flow dynamics with a validated method; and the hyperemic Pa (Pa_hyp_) was assumed by mean arterial pressure during the index procedure. Thus, caIMR was calculated as follows:

(1) $$\begin{array}{c} \text{caIMR}={\text{Pd}}_{\text{hyp}}\frac{L}{kV_{\text{diastole}}}, \end{array}$$

where *L* represents the length from the inlet to the distal position; Pd_hyp_ is the mean pressure at the distal position at the maximal hyperemia, which is computed by the software as the product of Pa_hyp_ and caFFR; *V*_diastole_ is the mean flow velocity at the distal position at diastole, which is derived using the cTFC method, and selection of the diastolic period is based on the movement of the tip of the guiding catheter; [12] and K is a constant (*K* = 2.1).

Two independently trained cardiologists who were blinded to clinical data and echocardiography results performed the analysis. Any contradictions were resolved by consensus.

### 2.3. Echocardiography Measurement and Analysis

Echocardiography was performed within the first 24 hours and at 3 months after PCI by two experienced cardiologists who were blinded to the clinical and coronary physiological information using an available ultrasound system (Vivid 7, GE Medical Systems, NY, USA). Left ventricular ejection fraction (LVEF) was measured from the four and two-chamber areas using the modified Simpson's rule. Wall motion score index (WMSI) was calculated according to the European society of echocardiography recommendations, using the 17-segment model on a 1–5 scale ((1) normal, (2) hypokinesia, (3) akinesia, (4) dyskinesia, and (5) aneurysmal) [18]. Global longitudinal strain (GLS) was assessed using speckle-tracking analysis and obtained from two-dimensional gray scale images of three standard apical views with optimal frame rate. Peak longitudinal strain was defined as the percent change in length of the myocardium from end-diastole to end-systole. The mean of the peak systolic longitudinal strain values from the 17 segments was calculated to determine GLS [19]. The change in LVEF, WMSI, and GLS was calculated by subtracting the baseline results from the follow-up ones. The definition of left ventricular recovery was an improvement in LVEF (i.e., the change in LVEF is more than zero) at 3 months.

### 2.4. Statistical Analysis

Statistical analysis was performed using the SPSS software (version 24.0, IBM Corp., NY, USA). Categorical variables were presented as frequency (%) and compared using the *χ*^2^ or Fisher's exact test, as appropriate. Continuous variables with normal distribution were presented as mean ± standard deviation, otherwise presented as median and interquartile range, which were compared using Student's *t*-test or Mann–Whitney *U* test, as appropriate. Bivariate correlation analysis was performed to assess the relationships between variables. The univariate logistic regression model was built and variables with *p* < 0.10 entered in the multivariate analysis. The factors that were deemed to be clinically relevant (age, sex, current smoking, and diabetes) were also incorporated. Then, we investigated the independent determinants of left ventricular recovery with a stepwise algorithm in the multivariate logistic regression analysis, and significant variables were included in the final predictive model. Similar to the method of risk score establishment proposed in Framingham' study [20], a model was developed by assigning weighted points for each variable, and a total score was calculated for each patient. Receiver operating characteristic (ROC) curve analysis was used to determine the best cutoff value and area under the curve (AUC) for the predictive model. The interobserver agreements for caIMR analysis were evaluated by calculating the intraclass correlation coefficients (ICC). A two-sided *p* value < 0.05 was considered to indicate a statistically significant difference.

## 3. Results

**Figure 1:**
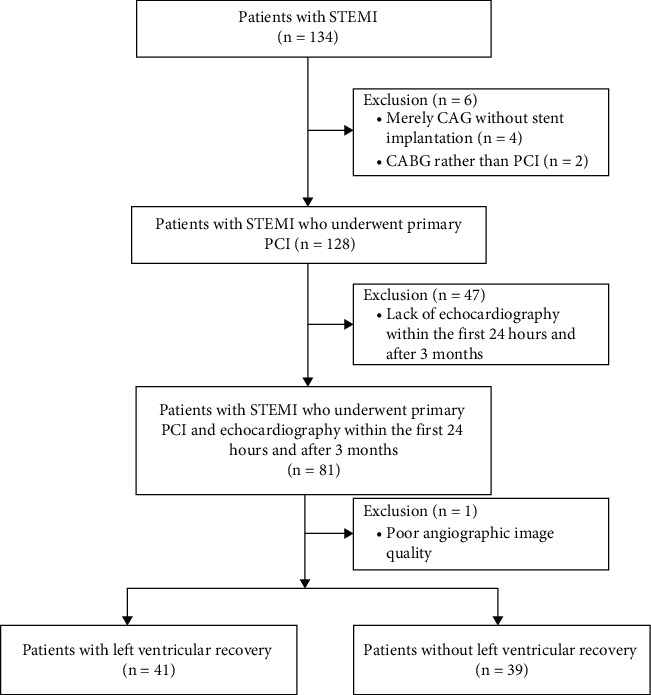
Study flowchart.

A total of 134 patients with STEMI who underwent primary PCI were screened for the present study. Of the 54 patients excluded, 2 required surgical revascularization, 4 received only coronary angiography, 47 lacked echocardiography within the first 24 hours or after 3 months, and in 1 patient, the angiographic image was unable to analyze due to poor quality. Thus, 80 patients were finally included (Figure 1).

The mean age of the patients was 58.0 ± 12.7 years. More than half of the patients had the coexisting risk factors of hypertension and smoking. The IRA was left anterior descending (LAD) in 51 patients, left circumflex in 8 patients, and right coronary in 21 patients. The time from symptom onset-to-balloon dilation was 7 (3.5–21.875) hours. Procedural success with TIMI flow grade 3 was achieved in 74 patients. Mean post-PCI caFFR and caIMR were 0.93 and 43.2, respectively. At discharge, all patients without contraindication were on therapy with aspirin, P2Y_12_ inhibitors, and statins, and most of the patients used *β*-blockers, angiotensin converting enzyme inhibitors, or angiotensin receptor blockers (Table 1).

The LVEF increased numerically at 3 months after the index procedure without significant difference compared to baseline. However, both WMSI (1.50 (1.24–1.88) vs 1.31 (1.08–1.63), *p* < 0.001) and GLS (−12.2 ± 4.0 vs −14.1 ± 4.0, *p* = 0.001) improved significantly at 3 months follow-up (Table 2). 41 of all patients showed left ventricular recovery. The mean post-PCI caIMR and cTFC were significantly lower in the patients with left ventricular recovery at 3 months (38.3 ± 15.5 vs. 48.4 ± 15.2, *p* = 0.004 and 20.2 ± 11.7 vs. 26.0 ± 13.7, *p* = 0.045, respectively). There was no significant difference in other physiological indices between the recovery and no recovery groups (Table 3).

**Figure 2:**
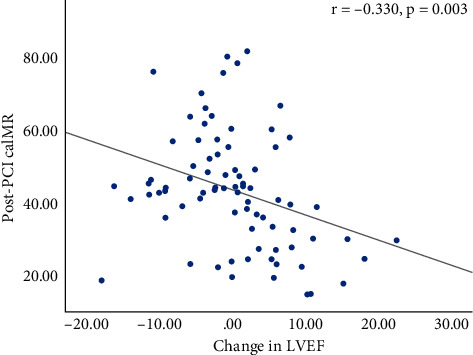
A significant inverse correlation found between coronary angiography-derived index of microcirculatory resistance after primary percutaneous coronary intervention and the change in left ventricular ejection fraction (*r* = −0.330, *p* = 0.003).

The post-PCI caIMR did not correlate with baseline and 3-month LVEF (*r* = 0.074, *p* = 0.512 and *r* = −0.169, *p* = 0.135, respectively). However, there was a significant inverse correlation between post-PCI caIMR and the change in LVEF (*r* = −0.330, *p* = 0.003, Figure 2). The change in LVEF, WMSI, and GLS all did not correlate with other measures of microvascular function.

In the univariate analysis, the peak CK-MB, post-PCI cTFC, and caIMR were significant predictors of left ventricular recovery at 3 months. The variables of age, sex, current smoking, hypertension, diabetes, hyperlipidemia, peak CK-MB, LAD as the culprit vessel, post-PCI cTFC, and caIMR were included for multivariate analysis. Then, the post-PCI caIMR (OR: 0.948, 95% CI: 0.916–0.981, *p* = 0.002), LAD as the culprit vessel (OR: 3.605, 95% CI: 1.23–10.567, *p* = 0.019), and male (OR: 0.254, 95% CI: 0.066–0.979, *p* = 0.047) were found to be independent predictors of left ventricular recovery in the multivariate logistic regression model (Table 4). The points were assigned based on regression coefficients, and we established a final predictive model as 0.054 × caIMR − 1.282 × LAD as the culprit vessel + 1.372 × male.

**Figure 3:**
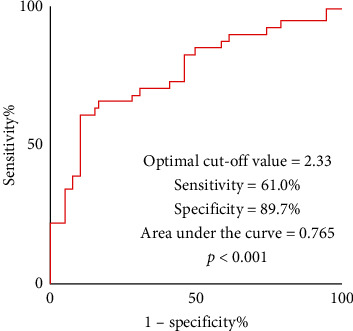
Receiver operating characteristic curves of the novel proposed predictive model. The optimal threshold of the model for predicting left ventricular recovery at 3-month follow-up was 2.33 with sensitivity 0.610, specificity 0.897, and area under the curve 0.765 (*p* < 0.001).

We then identified the optimal threshold for the prediction of left ventricular recovery by ROC curve analysis. The best cutoff value of the predictive model was 2.33 (sensitivity 0.610, specificity 0.897, AUC 0.765, 95% CI: 0.660–0.871, and *p* < 0.001) (Figure 3). The best cutoff value of the post-PCI caIMR alone was 40.9 with an AUC of 0.705.

Using 2.33 as the optimal cutoff value, 29 patients had a predictive model score less than 2.33. In patients with a predictive model score less than 2.33, the peak CK-MB, post-PCI cTFC, and caFFR were significantly lower. The proportion of multivessel disease was also significantly lower in these patients. There was no significant difference in the mean age, cardiovascular risk factors, blood pressure level, discharge medications, and ischemia time between the two groups (Table 5).

The LVEF increased significantly in patients with a predictive model score less than 2.33 (58.4 ± 11.3 vs 53.0 ± 12.8, *p* < 0.001), whereas it decreased significantly in the other group (55.7 ± 9.1 vs. 58.2 ± 9.8, *p* = 0.009). The WMSI was significantly lower at 3-month follow-up compared to baseline only in patients with a score more than or equal to 2.33 (1.31 (1.06–1.60) vs. 1.47 (1.21–1.94), *p* < 0.001). The GLS improved significantly in both the groups (−13.5 ± 4.3 vs. −11.5 ± 3.3, *p* = 0.015 and −14.4 ± 3.9 vs. −12.6 ± 4.3, *p* = 0.026, respectively) (Table 6). A significant difference was observed for the change in LVEF between the two groups (5.5 ± 6.6 vs. −2.5 ± 6.6, *p* < 0.001), while there was no difference for the change in WMSI and GLS (Table 5).

There was a good concordance between two cardiologists for the measurement of post-PCI caIMR of the culprit vessels (ICC = 0.889, *p* < 0.001).

## 4. Discussion

The present study examines the predictive value of postprocedural caIMR for left ventricular functional recovery in patients with STEMI who undergo primary PCI. The key findings of the present study are as follows: (i) the post-PCI caIMR of the IRA is an independent predictor of left ventricular functional recovery at 3 months after the index procedure and (ii) the female patients with lower post-PCI caIMR, in whom the culprit vessel is LAD, show a more significant improvement in left ventricular functional indices including LVEF and GLS.

Despite the success of primary PCI in IRA recanalization, approximately half of patients with STEMI show failure of myocardial reperfusion and CMD in the culprit vessel territory, which is a key determinant of adverse ventricular remodeling and clinical outcome [1]. Although many noninvasive imaging modalities are optimal for CMD assessment, they are not available at the cardiac catheterization laboratory during PCI [21]. The pressure wire-derived IMR measured immediately after PCI is a quantitative, reproducible index not affected by epicardial coronary artery stenosis under various hemodynamic perturbations and has been considered as a “gold standard” for CMD [2].

Overwhelming evidences suggest that IMR can accurately predict the size of myocardial infarction and remodeling of the left ventricle and microvascular obstruction in patients with STEMI treated by PCI [22–27]. Furthermore, IMR following PCI has the potential to predict left ventricular recovery at 3 months post-STEMI in patients managed with primary angioplasty and pharmacoinvasive strategies [3, 4]. It has also been found that the patients with a post-PCI mean IMR greater than 40 U have a higher rate of death or rehospitalization due to heart failure at 1 year in a multicenter study assessing 253 patients with STEMI [28].

However, due to the additional cost, extra procedural time, and risk associated with the manipulation of a pressure wire, patient discomfort caused by adenosine infusion, IMR has inevitable practical restrictions. Recently, some attempts have been made to calculate IMR based on coronary angiography without the need of a pressure wire and adenosine [5]. Tebaldi et al. proposed for the first time the formula of the angiography-based IMR, which shows a modest diagnostic performance for the prediction of IMR ≥ 25 [6]. Mejia–Renteria et al. developed a method applicable to functional angiography and demonstrated that estimation of IMR without physiological wires and adenosine is feasible [7]. Ai et al. confirmed the high diagnostic accuracy of caIMR using IMR as the reference standard in patients with ischemia and no obstructive coronary arteries [8]. Similarly, De Maria et al. validated that angiography-derived IMR is a promising alternative of invasive IMR to detect CMD in patients with STEMI and stable coronary artery disease [9, 10]. As a novel tool for the assessment of CMD, the pooled sensitivity and specificity of angiography-based IMR are 0.81 and 0.80, respectively, regardless of patients' presentation in a meta-analysis [11].

Consistent with previous studies, the post-PCI caIMR shows a great capability for the prediction of left ventricular functional recovery at 3 months in the present study, and the best cutoff value for caIMR is close to the well-established threshold of IMR to define CMD in STEMI [28]. In addition, the angiography-derived IMR after PCI has been confirmed as an independent predictor of long-term clinical outcome including cardiac death or readmission for heart failure in patients with STEMI and stable coronary artery disease [12–15]. The culprit vessel is LAD has been another independent predictor of left ventricular performance recovery in the present study, suggesting that the successful recanalization of LAD in STEMI treated with PCI is extremely important for the cardiac function.

The present study has several limitations. First, the study is retrospective with a relatively small sample size. Thus, the established predictive model is not a real prospective prediction rather than only an internal validation. Second, our included patients fail to receive IMR measurements as a reference. Third, CMD in the IRA territory can be a dynamic phenomenon; therefore, a single caIMR value immediately after PCI might not fully explain patient prognosis. Next, there is a lack of evaluation of the underlying CMD in the nonculprit vessel territory. Finally, the clinical prognostic implication of caIMR has not been assessed.

## 5. Conclusions

The novel calculated caIMR after primary PCI shows a great value for the prediction of left ventricular functional recovery reflected by LVEF improvement at 3 months after the index procedure in patients with STEMI.

## Figures and Tables

**Table 1:** Clinical, angiographic, and procedural characteristics.

Variable	Total (*n* = 80)
Demographics	
Age (y)	58.0 ± 12.7
Male	64 (80.0%)
Body mass index (kg/m^2^)	24.7 ± 3.3
Cardiovascular risk factors	
Hypertension	50 (62.5%)
Diabetes	26 (32.5%)
Hyperlipidemia	39 (48.8%)
Chronic kidney disease	4 (5.0%)
Current smoking	47 (58.8%)
Previous PCI	7 (8.8%)
Previous CABG	1 (1.3%)
Hemodynamic parameters	
Systolic blood pressure (mmHg)	125.5 ± 19.4
Diastolic blood pressure (mmHg)	72.7 ± 10.4
Cardiac function (Killip class)	
I	65 (81.3%)
II	8 (10.0%)
III	1 (1.3%)
IV	6 (7.5%)
Culprit vessel	
Left anterior descending	51 (63.7%)
Left circumflex	8 (10.0%)
Right coronary artery	21 (26.3%)
Multivessel disease	61 (76.3%)
Symptom onset-to-balloon time (h)	7 (3.5–21.875)
Thrombus aspiration	7 (8.8%)
Glycoprotein IIb/IIIa inhibitor use	8 (10.0%)
Adjunctive balloon dilatation	5 (6.3%)
Total number of stents	1 (1-2)
Mean stent diameter (mm)	3.0 (2.75–3.5)
Total length of stents (mm)	32 (24–46)
Post-PCI TIMI flow grade	
2	6 (7.5%)
3	74 (92.5%)
Coronary physiological measurements	
cTFC	22.98 ± 13.00
caFFR	0.93 (0.89–0.94)
caIMR	43.2 ± 16.1
Laboratory profiles	
Peak troponin I (ng/ml)	19742.35 (81.00–74380.48)
Peak CK-MB (ng/ml)	180.79 (56.02–238.48)
C-reactive protein (mg/L)	8.8 (1.0–30.3)
Low-density lipoprotein cholesterol (mmol/L)	2.88 ± 0.87
Estimated glomerular filtration rate (ml/min*∗*1.73 m^2^)	87.4 ± 20.6
Discharge medication	
Aspirin	80 (100%)
P2Y_12_ inhibitor	80 (100%)
Statins	80 (100%)
Beta-blocker	66 (82.5%)
ACEI/ARB	60 (75.0%)

PCI, percutaneous coronary intervention; CABG, coronary artery bypass graft; TIMI, thrombolysis in myocardial infarction; cTFC, corrected thrombolysis in myocardial infarction frame count; caFFR, coronary angiography-derived fractional flow reserve; caIMR, coronary angiography-derived index of microcirculatory resistance; ACEI, angiotensin converting enzyme inhibitor; ARB, angiotensin receptor blocker.

**Table 2:** Baseline and 3 months echocardiographic parameters.

Parameter	Baseline	3 months	*P* value
LVEF	56.3 ± 11.2	56.7 ± 10.0	0.661
WMSI	1.50 (1.24–1.88)	1.31 (1.08–1.63)	<0.001
GLS	−12.2 ± 4.0	−14.1 ± 4.0	0.001

LVEF, left ventricular ejection fraction; WMSI, wall motion score index; GLS, global longitudinal strain.

**Table 3:** Measures of microvascular function in patients with and without left ventricular recovery at 3 months.

Variable	Recovery (*n* = 41)	No recovery (*n* = 39)	*P* value
TIMI flow grade 3	39 (95.1%)	35 (89.7%)	0.625
cTFC	20.2 ± 11.7	26.0 ± 13.7	0.045
caFFR	0.92 (0.90–0.945)	0.93 (0.88–0.93)	0.227
caIMR	38.3 ± 15.5	48.4 ± 15.2	0.004

TIMI, thrombolysis in myocardial infarction; cTFC, corrected thrombolysis in myocardial infarction frame count; caFFR, coronary angiography-derived fractional flow reserve; caIMR, coronary angiography-derived index of microcirculatory resistance.

**Table 4:** Logistic regression analysis for the left ventricular recovery at 3 months.

Univariable	Odds ratio	95% CI	*P* value
Age	1.018	0.983–1.055	0.316
Male	0.401	0.125–1.287	0.124
Hypertension	0.455	0.180–1.152	0.097
Diabetes	0.929	0.364–2.367	0.877
Hyperlipidemia	0.445	0.182–1.089	0.076
Current smoking	0.525	0.212–1.297	0.163
Cardiac function (Killip class)	0.975	0.578–1.645	0.926
Peak CK-MB	0.995	0.990–1.000	0.047
Multivessel disease	0.704	0.249–1.991	0.508
LAD as the culprit vessel	2.338	0.918–5.953	0.075
Post-PCI TIMI flow grade	2.229	0.384–12.923	0.372
cTFC	0.964	0.930–1.000	0.050
caFFR	0.016	0–16.669	0.243
caIMR	0.957	0.927–0.988	0.007
Multivariable	Odds ratio	95% CI	*P* value
Male	0.254	0.066–0.979	0.047
LAD as the culprit vessel	3.605	1.230–10.567	0.019
caIMR	0.948	0.916–0.981	0.002

LAD, left anterior descending; TIMI, thrombolysis in myocardial infarction; cTFC, corrected thrombolysis in myocardial infarction frame count; caFFR, coronary angiography-derived fractional flow reserve; caIMR, coronary angiography-derived index of microcirculatory resistance.

**Table 5:** Clinical variables and echocardiographic parameters of patients according to the optimal cutoff value for the predictive model.

Variable	The score < 2.33 (*n* = 29)	The score ≥ 2.33 (*n* = 51)	*P* value
Age (y)	60.3 ± 15.6	56.7 ± 10.7	0.274
Body mass index (kg/m^2^)	24.4 ± 3.9	24.9 ± 2.9	0.453
Hypertension	15 (51.7%)	35 (68.6%)	0.133
Diabetes	7 (24.1%)	19 (37.3%)	0.229
Hyperlipidemia	12 (41.4%)	27 (52.9%)	0.320
Current smoking	16 (55.2%)	31 (60.8%)	0.624
Systolic blood pressure (mmHg)	125.1 ± 19.6	125.8 ± 19.5	0.888
Diastolic blood pressure (mmHg)	69.8 ± 11.0	74.4 ± 9.8	0.058
Multivessel disease	18 (62.1%)	43 (84.3%)	0.025
Symptom onset-to-balloon time (h)	8.0 (4.5–24.5)	6.0 (3.5–16.2)	0.312
Thrombus aspiration	0 (0.0%)	7 (13.7%)	0.094
Glycoprotein IIb/IIIa inhibitor use	2 (6.9%)	6 (11.8%)	0.756
Post-PCI TIMI flow grade 3	27 (93.1%)	47 (92.2%)	0.877
cTFC	17.6 ± 9.0	26.0 ± 14.0	0.002
caFFR	0.905 (0.83–0.925)	0.93 (0.91–0.945)	0.001
Peak troponin I (ng/ml)	9313.0 (40.9–54053.55)	25682.9 (82.0–91652.2)	0.091
Peak CK-MB (ng/ml)	80.9 (31.36–226.65)	194.0 (104.3–243.4)	0.039
Beta-blocker	23 (79.3%)	43 (84.3%)	0.571
ACEI/ARB	23 (79.3%)	37 (72.5%)	0.502
Change in LVEF	5.5 ± 6.6	−2.5 ± 6.6	<0.001
Change in WMSI	−0.05 (−0.25–0)	−0.16 (−0.47–−0.03)	0.165
Change in GLS	−2.4 ± 3.4	−1.8 ± 3.8	0.610

TIMI, thrombolysis in myocardial infarction; cTFC, corrected thrombolysis in myocardial infarction frame count; caFFR, coronary angiography-derived fractional flow reserve; caIMR, coronary angiography-derived index of microcirculatory resistance; ACEI, angiotensin converting enzyme inhibitor; ARB, angiotensin receptor blocker; LVEF, left ventricular ejection fraction; WMSI, wall motion score index; GLS, global longitudinal strain.

**Table 6:** Baseline and 3-month echocardiographic parameters according to the optimal cutoff value for the predictive model.

Parameter	The score < 2.33			The score ≥ 2.33		
	Baseline	3 months	*P* value	Baseline	3 months	*P* value
LVEF	53.0 ± 12.8	58.4 ± 11.3	<0.001	58.2 ± 9.8	55.7 ± 9.1	0.009
WMSI	1.50 (1.31–1.78)	1.38 (1.13–1.63)	0.077	1.47 (1.21–1.94)	1.31 (1.06–1.60)	<0.001
GLS	−11.5 ± 3.3	−13.5 ± 4.3	0.015	−12.6 ± 4.3	−14.4 ± 3.9	0.026

LVEF, left ventricular ejection fraction; WMSI, wall motion score index; GLS, global longitudinal strain.

## Data Availability

The datasets used and/or analyzed during the current study are available from the corresponding author upon request.
